# MicroRNAs differentially present in the plasma of HIV elite controllers reduce HIV infection *in vitro*

**DOI:** 10.1038/srep05915

**Published:** 2014-08-01

**Authors:** Rita Reynoso, Natalia Laufer, Matthias Hackl, Susanna Skalicky, Rossella Monteforte, Gabriela Turk, Mauricio Carobene, Jorge Quarleri, Pedro Cahn, Roland Werner, Heribert Stoiber, Regina Grillari-Voglauer, Johannes Grillari

**Affiliations:** 1 Department of Biotechnology, CD laboratory on Biotechnology of Skin Aging, BOKU - University of Natural Resources and Life Sciences Vienna, Muthgasse 18, A-1190 Vienna, Austria; 2 Instituto de Investigaciones Biomédicas en Retrovirus y SIDA (INBIRS), Universidad de Buenos Aires/CONICET, Buenos Aires, Argentina; 3 CONICET, Argentina; 4 TAmiRNA GmbH, Muthgasse 11, A-1190 Vienna, Austria; 5 Evercyte GmbH, Muthgasse 18, A-1190 Vienna, Austria; 6 J.A. Fernández Hospital, Infectious Diseases Unit, Buenos Aires, Argentina; 7 Huesped Foundation, Buenos Aires, Argentina; 8 Division of Virology, Innsbruck Medical University, Innsbruck, Austria

## Abstract

Elite controllers maintain HIV-1 viral loads below the limit of detection. The mechanisms responsible for this phenomenon are poorly understood. As microRNAs (miRNAs) are regulators of gene expression and some of them modulate HIV infection, we have studied the miRNA profile in plasma from HIV elite controllers and chronically infected individuals and compared against healthy donors. Several miRNAs correlate with CD4+ T cell count or with the known time of infection. No significant differences were observed between elite controllers and healthy donors; however, 16 miRNAs were different in the plasma of chronic infected versus healthy donors. In addition, levels of hsa-miR-29b-3p, hsa-miR-33a-5p and hsa-miR-146a-5p were higher in plasma from elite controllers than chronic infected and hsa-miR-29b-3p and hsa-miR-33a-5p overexpression significantly reduced the viral production in MT2 and primary T CD4+ cells. Therefore, levels of circulating miRNAs might be of diagnostic and/or prognostic value for HIV infection, and hsa-miR-29b-3p and miR-33a-5p may contribute to the design of new anti-HIV drugs.

Infection with HIV is characterized by a progressive decrease of CD4+ T lymphocytes and immune dysfunctions that ultimately lead to AIDS. However, between 1% to 5% of people infected with HIV, known as long-term nonprogressors: (LTNP), are able to maintain stable CD4+T cell counts, showing important variations in their viremia levels^1^,^2^. Furthermore, a rare subgroup among these LTNP, termed “elite suppressors” or “elite controllers” (EC), maintain plasma viral loads below the limit of detection of commercial assays (50 copies/ml) and usually do not show any clinical signs of disease progression for many years^3^,^4^.

Several studies have shown that not only viral characteristics such as mutations or deletions in viral proteins like Gag, Nef, and other accessory proteins were associated to a disease control^5^,^6^,^7^, but also that host specific immunological determinants and genetic background are related to elite suppression of viral replication^8^, since replication-competent viruses have been isolated from EC^9^,^10^. Examples of such genetic differences include specific expression of HLA class I complex, mutations in the gene for the human chemokine receptor 5 (CCR5) or the functionality of T CD8+ lymphocytes^11^,^12^,^13^. Nevertheless, it is unlikely that only these variations can explain the remarkable immune response of EC against HIV infection.

Interestingly, Witwer et al. recently revealed notable similarities in microRNA (miRNA) profiles between peripheral mononuclear cells (PBMC) from EC and healthy donors when compared to viremic HIV patients^14^. This suggests that intracellular miRNAs might be implicated in the particular antiretroviral-free control of HIV infection. MiRNAs are small (18–24 bp) non-coding RNAs, which have important regulatory roles in the cells by silencing mRNA expression through the interaction with the RNA-induced silencing complex (RISC), resulting in mRNA cleavage or translational repression^15^. The ability of miRNAs to bind and repress the translation of up to hundreds of mRNAs establishes miRNAs as central regulators of gene expression with important biological roles including the regulation of antiviral defenses^16^. In addition, miRNAs that are encapsulated by microvesicles or bound to proteins can exit immune cells or other tissues and circulate the blood stream^17^. Interestingly, the concentration profile of miRNAs in plasma/serum has been proposed as a useful tool for diagnosis and prognosis of several diseases including cancer or cardiovascular diseases^18^,^19^,^20^,^21^.

Consequently, we hypothesized that a specific signature of miRNAs in plasma/serum might discriminate elite controllers from patients with chronic HIV infection. In addition, since it has been shown that HIV replication can be modulated by the expression of human miRNAs^22^,^23^, we also hypothesized that such miRNAs might be involved in controlling HIV infection. This assumption seemed conceivable, since miRNAs are also known to be transferred to recipient cells, where they are actively controlling gene expression^17^.

Thus, we set out to profile 175 miRNAs in plasma derived from EC, chronic HIV progressors (CH) and healthy donors (HD). Thereby, we discovered 16 differentially expressed miRNAs between CH and HD, and 3 between CH and EC. We then tested, if these latter three miRNAs influence the replication of HIV in lymphocytes as *in vitro* model system, and indeed observed that hsa-miR-29b-3p and hsa-miR-33a-5p overexpression significantly reduced virus production. Finally, levels of these miRNAs in plasma samples from treatment-naive HIV infected patients were analysed to find a similar response as in chronic HIV patients undergoing anti-retroviral treatment (ART). Therefore, we suggest that plasma derived miRNA signatures might be of prognostic value for HIV, but also for viral infections in general. In addition, hsa-miR-29b-3p and miR-33a-5p might be used in developing therapeutic strategies against HIV.

## Results

### Donor characteristics

We studied the expression levels of circulating miRNAs in EDTA-plasma samples derived from 27 subjects, 10 CH, 10 EC and 7 HD. Stringent quality criteria were applied in order to avoid bias introduced during sample preparation including RNA isolation, cDNA synthesis and qPCR (see Material and Methods, Supplemental figure 1). Consequently, 9 samples from EC, 9 CH and 6 HD were included in the analysis. One sample from the CH group was excluded from the analysis due to a very specific liver toxicity profile, which we regarded as confounding to our aims. This liver toxicity is explained by co-infection of the patient with Hepatitis B virus and was detected due to high levels of hsa-miR-194 and hsa-miR-122 as described^24^,^25^ (Supplemental figure 1d).

The characteristics of the donors included in this study are summarized in Table 1. There were no differences between the groups regarding age and gender (p = 0.790 and p = 0.862 respectively). CD4+ T cell counts and the known time of infection did not statistically differ between EC and CH (p = 0.298 and p = 0.722 respectively). The viral load was below 50 copies/ml in all the samples of the EC group and in 8 of the CH group, in which one mounted to 568 copies/ml.

### MiRNA plasma profiles discriminate chronically infected patients

**Figure 1:**
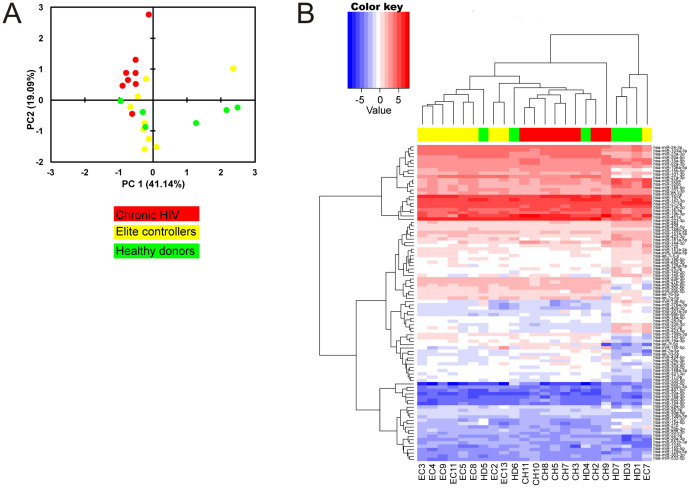
Explorative data analysis. (A) Principal components analysis of miRNAs profile in plasma was done on all miRNAs. The normalized Cp values were used for the analysis. The x-axis corresponds to principal component 1 (PC1) and the y-axis to the principal component 2 (PC2), the percentage of the variance is indicated between brackets. (B) Heatmap and hierarchical clustering. The clustering is performed on all the samples and the 96 miRNAs expressed in all the samples, using complete linkage and Euclidean distance as distance metric. Each column represents one sample and each row one miRNA. The miRNA clustering tree is shown on the left. The color-coded scale (blue: expression levels lower than the mean and red: expression level over the mean) for the normalized expression value is indicated at the top of the figure.

In total 175 miRNAs were analyzed in this study using the miRCURY LNA™ Serum/Plasma Focus PCR panel. On average, 153 miRNAs were detected per sample and 96 miRNAs gave reliable signals in all 23 samples (Supplemental File 1). In the first step, an exploratory data analysis was performed in order to evaluate grouping of studied samples according to their disease state and to identify co-regulated sets of miRNAs. Therefore, a principal component analysis (PCA) was performed with the intention of reducing the dimensionality of the data set from 175 miRNAs to two dimensions (PC1 and PC2), accounting for more than 60% of the variability in the data set. Indeed, PCA revealed that EC and HD have similarities, and that they both are clearly separated from CH samples, indicating that miRNAs might be valuable discriminators between the groups. This was supported by unsupervised hierarchically clustering using Euclidean distance and complete linkage analysis based on the set of 96 miRNAs present in all samples. Thereby, the majority of EC fall together into one cluster and are clearly separated from the cluster containing samples from CH (Figure 1b). Samples representing HD partly cluster with EC samples or are represented in an independent cluster to the right. Overall, these data strongly suggest that miRNA profiles in plasma samples from EC are different from the observed in patients with chronic HIV infection and, in some instances, similar to that of HD.

### MiRNA levels are altered in plasma of HIV patients

**Figure 2:**
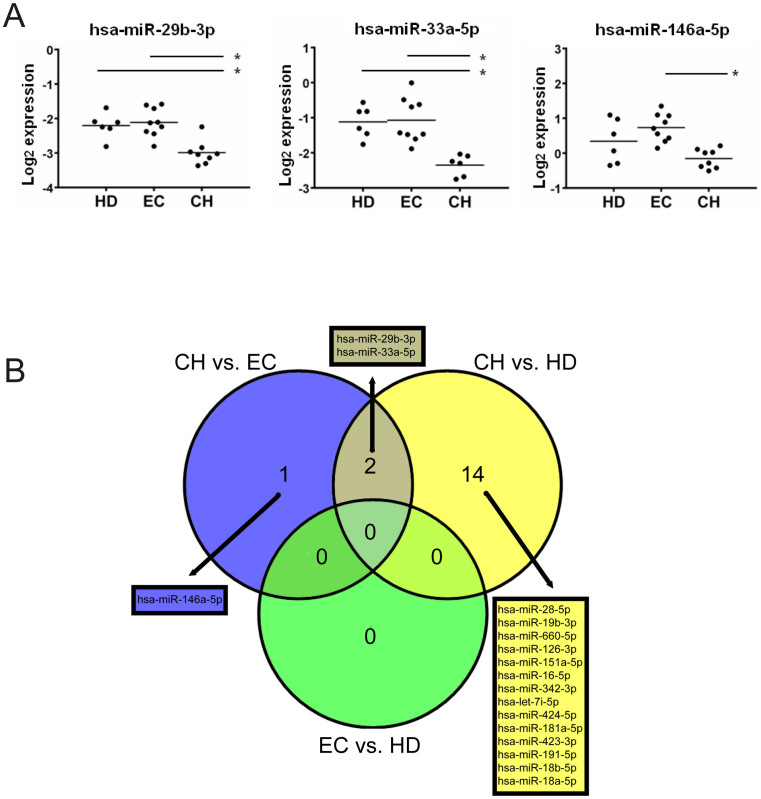
Differentially-expressed miRNAs. (A) Dot plot showing miRNAs differentially expressed between EC and CH. Each dot indicates the individual level of expression and the line is representing the mean of the group. Asterisks indicate a q value ≤ 0.05. (B) Venn diagram showing the overlapping of the differentially expressed miRNAs. HD: healthy donors, EC: elite controllers, CH: chronic HIV.

To further identify differentially expressed miRNAs between the groups, we compared the relative miRNA expression (dCp) applying ANOVA followed by post-hoc Bonferroni test. We found 49 miRNAs differentially expressed between the groups, suggesting that HIV infection strongly influences the plasma miRNA profile (Supplemental table 1). After correction for false positive discovery rate (FDR) in multiple comparisons using the Hochberg test, 16 miRNAs passed the p-value adjustment and were identified significantly different between CH and HD (Table 2). In addition, plasma levels of hsa-miR-29b-3p, hsa-miR-33a-5p and hsa-miR-146a-5p were different between CH and EC (Figure 2a). As shown in the Venn diagram hsa-miR-29b-3p and hsa-miR-33a-5p were also found differentially expressed between CH and HD, while no significant differences were observed between EC and HD (Figure 2b). Next, we wanted to test whether the expression of these miRNAs is also modified in plasma from patients with progressive disease and without ART. Therefore, 20 plasma samples from untreated patients (PVL mean: 124077 ± 146560 copies/ml, CD4+ T cell count mean: 108 ± 102 cells/μl) were obtained and the levels of circulating hsa-miR-29b-3p, hsa-miR-33a-5p and hsa-miR-146a-5p were analyzed by qPCR. Again the expression levels of hsa-miR-29b-3p and hsa-miR-33a-5p were significantly lower in samples from patients with HIV comparing to HD (p = 0.0413 and p = 0.0014 respectively) (Supplemental Figure 2).

Together, these results indicate that HIV infection influences levels of specific miRNAs in the blood, which is independent of ART in case of hsa-miR-29b-3p and hsa-miR-33a-5p.

### Specific miRNAs in plasma correlate with T CD4+ cell count and the known time of infection

**Figure 3:**
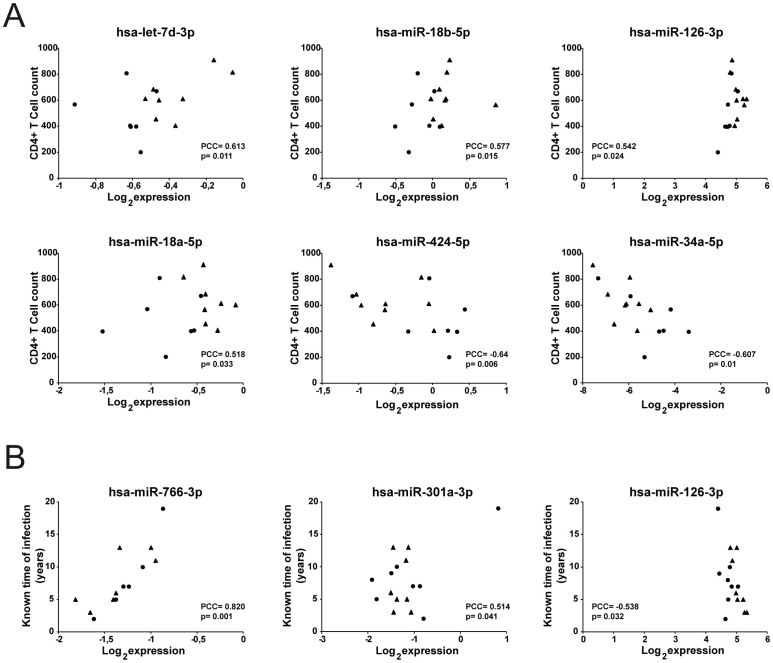
miRNAs correlated with T CD4+ cell count and known time of infection. (A) Six miRNAs were found to be correlated with T CD4+ cell count. (B) Three miRNAs were correlated with known time of infection in years. The analysis was performed considering all the HIV infected samples. PCC: Pearson correlation coefficient, p: p value. The p values indicate the significance of the Pearson correlation. •: chronic HIV, ▴: elite controllers.

After discovering differences in the miRNA plasma profile between the groups, we were interested in finding out relations between the levels of miRNA expression in plasma and specific clinical parameters. Interestingly, we found that hsa-miR-18b-5p, hsa-miR-126-3p, hsa-let-7d-3p and hsa-miR-18a-5p correlated positively with CD4+ T cell counts (p = 0.015, p = 0.024, p = 0.011, and p = 0.033 respectively). On the other hand, hsa-miR-424-5p and hsa-miR-34a-5p correlated negatively (p = 0.006 and p = 0.01 respectively; Figure 3a). In addition, we observed that hsa-miR-766-3p and hsa-miR-301a-3p correlate positively and hsa-miR-126-3p negatively with the known time of infection (Figure 3b), whereby the correlation of miR-766-3p is the most striking as it linearly increases over time. Despite the small sample size, these observations strongly suggest that the expression levels of specific miRNAs are related to CD4+ T cell count and additionally the levels of some miRNAs would be modified during the progression of HIV infection.

### Hsa-miR-29b-3p and hsa-miR-33a interfere with HIV replication *in vitro*

**Figure 4:**
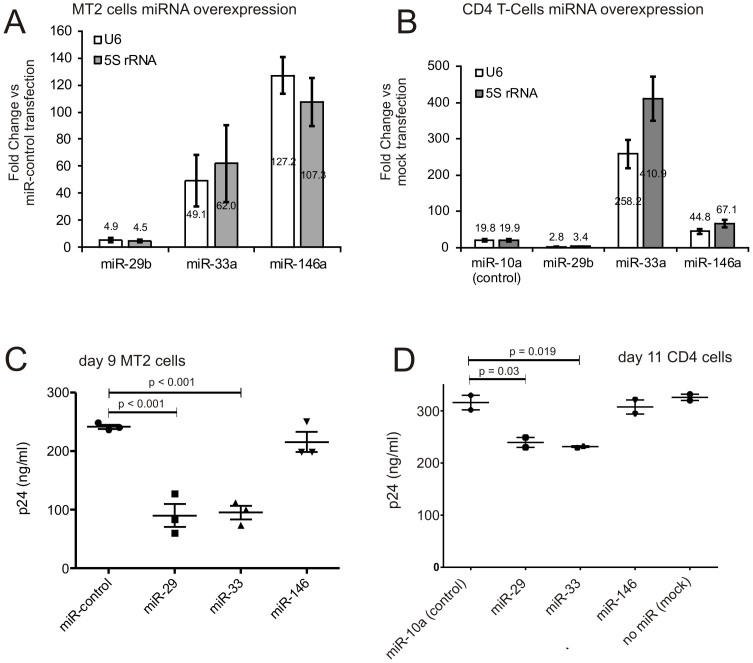
miRNAs interfere with HIV replication. (A) Real-time qPCR analysis of hsa-miR-29b-3p, hsa-miR-33a-5p and hsa-miR-146a was performed 48 hours post transfection in MT2 cells. Two independent reference genes (U6 and 5S rRNA) were used for normalization and fold changes in miRNA levels relative to a scrambled negative control miRNA (fold-change = 1, not shown) were calculated as average from 3 independent transfections (+/−SD). (B) Analogous qPCR data for CD4 cells. hsa-miR-10a was included in this experiment as non-functional negative control, since it was not differentially present in plasma samples of CH, EC and HD. (C) Forty eight hours after transfection MT2 cells were infected with HIV-NL4.3. p24 antigen titers are given in ng/ml at day 9 post infection of MT2 cells. (D) p24 antigen titers from supernatant of primary CD4-positive T cells at day 11 after infection with HIV. As shown, the overexpression of hsa-miR-29b-3p and hsa-miR-33a-5p reduce significantly the production of p24 antigen (MT2 cells: hsa-miR-29b-3p p < 0.001 and hsa-miR-33a-5p p < 0.001; primary CD4-positive cells: hsa-miR-29b-3p p = 0.03 and hsa-miR-33a-5p p = 0.019). Three independent infections after miRNA or scrambled-control transfections were performed and the respective p24 titers are shown for each infection as average of two technical replicates.

Since several reports have shown that circulating miRNAs can be functionally transferred to recipient cells, we tested, if the 3 differentially expressed miRNAs in plasma of EC versus CH (down-regulated in CH) might influence HIV replication *in vitro*. Therefore, we pre-transfected these miRNAs and a scrambled non-targeting control miRNA into MT2 as model for T-cells and subsequently into primary CD4 T cells, where hsa-miR-10a was used as putative non-functional control, since it was not found to be differentially present in plasma samples, together with non-infected cells (Figure 4). Transfection efficiency in CD4 cells was controlled using Cy-3 labelled miRNA mimics (Supplemental Figure 3) and microRNA overexpression was evaluated 48 hours post transfection by qPCR (Figure 4 A–B). After miRNA transfection, cells were in turn infected with HIV-NL4.3. The viral replication was quantified by ELISA of p24 in cell supernatants over 9 days (MT2) or 11 days (CD4) (Figure 4 C,D).

Since hsa-miR-146a-5p is known to down-regulate the CXCR4 receptor involved in infection by HIV (26) we tested this miRNA first. However, no inhibition of HIV infection was observed. When testing CXCR4 expression after miR-146-5p transfection in MT2 cells by flow cytometry, no reduction of CXCR4 was observed (Supplemental Table 2), potentially accounting for its lack of inhibitory activity on HIV infection. In contrast, the other two miRNAs, hsa-miR-29b-3p as well as hsa-miR-33a-5p significantly decreased p24 in the cellular supernatants as compared to non-targeting control transfected cells (MT2 cells: hsa-miR-29b-3p p < 0.001 and hsa-miR-33a-5p p < 0.001; primary CD4-positive cells: hsa-miR-29b-3p p = 0.03 and hsa-miR-33a-5p p = 0.019) (Figure 4 C,D). Combining hsa-miR-29b-3p and hsa-miR-33a-5p had no synergistically inhibitory effect on the viral replication (data not shown). These results suggest that miRNAs present in the blood of elite controllers at higher levels than in chronically diseased individuals might contribute to a successful defence against HIV progression to AIDS.

## Discussion

Here we describe differences in the plasma miRNA signatures of HIV infected individuals. We show that 16 miRNAs are differentially expressed between CH and HD. Interestingly, some of these miRNAs have been previously reported to play a role either in HIV infection or in the functionality of the immune system. For example, hsa-miR-28-5p belongs to the cluster of cellular anti-HIV miRNAs that induce the viral latency in CD4+ T cells and also inhibits viral replication in monocytes/macrophages^22^,^23^,^26^. In addition, hsa-miR-181a-5p is associated to the regulation of T cell development and inflammatory responses of monocytes/macrophages^27^,^28^. Therefore, the down-regulation of these miRNAs by HIV might be one of the mechanisms associated to disease progression.

But where do these miRNAs in the plasma come from? Previous investigations of blood cells indicate that HIV infection -both *in vivo* and *in vitro*- affects the expression of miRNAs in various immune cells^14^,^29^,^30^,^31^,^32^, and that these cells are able to secrete miRNAs depending on various conditions^33^,^34^. This would suggest that blood cells are an active source of secretion, especially in the case of let-7d-3p, miR-18a-5p, miR-18b-5p, miR-126-3p, and miR-424-5p that correlate positively with T CD4+ cell count. One miRNA, however, miR-34a-5p is elevated in plasma when CD4 T cell counts get lower. Since miR-34a-5p has been found to be high in CD4+ T cells in EC^14^, the negative correlation between T CD4+ and plasma miR-34a-3p levels might indicate that miR-34a-3p ends up in the circulation due to CD4+ T cell lysis. However, as miRNAs present in plasma can derive from several cell types^17^,^35^, more work is necessary to identify which miRNAs might originate from which cell types.

We also find that some miRNAs correlate to the known time of infection independently of CH or EC status of the patients. This suggests that in CH the expression of plasma miRNAs might be useful in predicting the disease progression and that the fight against the virus adapts the organismal environment in a way that is mirrored by the circulating miRNAs. It is unclear, why EC display a similar change in circulating miRNAs. Therefore, more studies, especially longitudinal studies, are necessary to establish the clinical relevance of these findings.

Finally, the plasma miRNA profile can discriminate between EC and CH. We show that the expression levels of hsa-miR-29b-3p, hsa-miR-33a-5p and hsa-miR-146a-5p are higher in plasma from EC than CH. This is in accordance to recent studies where intracellular miRNA profiles in PBMC were found to discriminate EC and CH. One miRNA, miR-29b, is found in both, PBMC and plasma^14^, as well as the orthologous miRNA to the here described miR-146a-5p, namely miR-146b-5p^36^. All other PBMC derived miRNAs, miR-125b, miR-150 and miR-31^14^ and miR-155^32^ did not reach significance in our setting.

In conclusion, the finding that the levels of hsa-miR29-3p, hsa-miR-33a-5p and hsa-miR-146a-5p are higher in the plasma of EC than in CH is striking, especially, if considering that miR-29b-3p is known to target Nef^37^,^38^,^39^ and alterations in Nef functions have been associated to slower progression to AIDS^40^,^41^. Similarly, hsa-miR-146a-5p has been found to target CXCR4^42^ and thus it could affect HIV infection, while no connection of miR-33a and HIV infection has been published so far. Since miRNAs that are found in plasma can be taken up by immune cells^35^,^43^, we were intrigued by the possibility that these miRNAs might also modify infection by HIV in our setting. Indeed, miR-33a-5p and miR-29b-3p reduced the production of p24 *in vitro*, while miR-146a-5p had no influence. This lack of miR-146a-5p activity was mirrored by the fact that CXCR4 was not knocked-down by in response to its overexpression under the conditions used here. Therefore more work is needed to clarify whether miR-146a-5p has an effect in the immune response or if it might interfere with HIV replication *in vivo* by targeting CXCR4 as important co-receptor of the HIV entry route into T cells.

**Figure 5:**
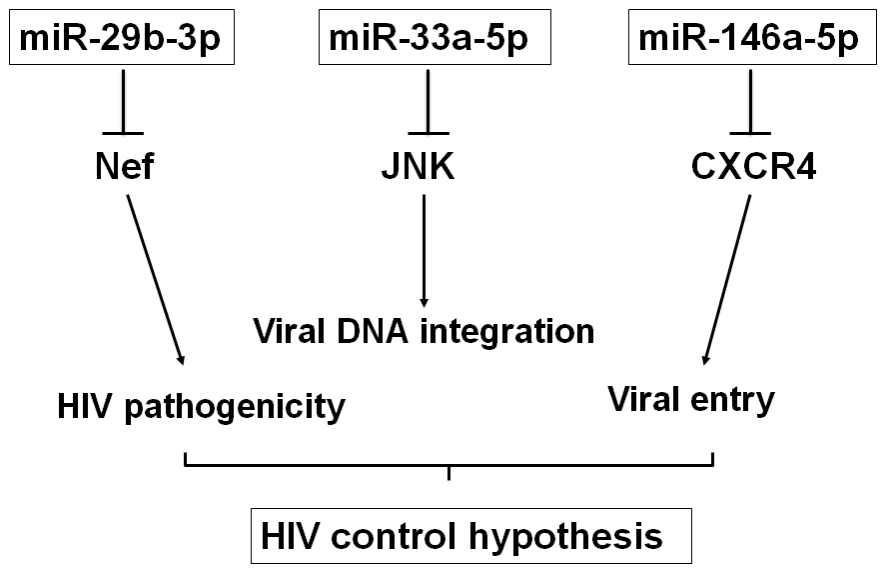
MiRNAs differentially expressed between EC and CH target genes with important functions in HIV cycle. Hypothetical figure showing how hsa-miR29b-3p, hsa-miR33a-5p and hsa-miR146a-5p could affect the replication of HIV.

How might the activity of miR-33a-5p be explained? We speculate that the effect may be mediated by inhibition of MAPK8 (also known as JNK), which is a validated target of this miRNA^44^ and described to phosphorylate the viral integrase favouring the enzyme stability and consequently an efficient integration of the proviral DNA^45^. These data would suggest that all three miRNAs might be part of a ‘native’ anti-viral defence by targeting important steps during HIV infection (Figure 5), since they are all found at higher levels in plasma of HD and ECs. Their downregulation in CH might therefore either simply be a consequence of the loss and premature aging of the immune system, or an active mechanism of HIV to suppress the anti-viral defence of the host.

Summarized, we propose that plasma miRNA profiling might be used as diagnostic or prognostic markers in HIV pathogenesis and disease progression. Moreover, hsa-miR-29b-3p and hsa-miR-33a-5p, or mimicks thereof, might have the potential to be used in therapeutic strategies against HIV infection and AIDS.

## Methods

### Study population

Plasma samples were obtained from 27 subjects and were classified in 3 groups, 10 Elite Controllers (EC), 10 chronic HIV patients (CH) under ART and 7 healthy donors (HD) The group of the EC was defined as individuals with plasma viral load (PVL) < 50 copies/ml, CD4 count > 350/ml. The PVL from EC patients remain undetectable during the follow up period and CD4 cell counts were stable for a period of at least 5 years. None of the patients developed opportunistic infections or HIV associated conditions. The criteria for including patients in the group with progressive disease were that patient reached a CD4 cell count lower than 350 cells/μL and/or developed opportunistic infections. The patients have been on treatment for a mean time of 5 years [(Interquartile range (IQR) 2.5–8.5 years)] and were aviremic for a mean time of 4.9 years (IQR 2.5–8). All of treated patients received reverse transcriptase inhibitors, 50% received zidovudine, 90% lamivudine, 22% tenofovir, 22% abacavir, 66% non-nucleoside reverse transcriptase inhibitors, 22% protease inhibitors. In a second step, 20 patients with progressive HIV disease but without ART were recruited. All the groups were chosen from the same population.

This study was approved by the Huésped Foundation Ethics Committee and informed consent was obtained from all subjects. All experiments within this study were performed in accordance with the Declaration of Helsinki.

### Isolation of RNA from plasma

EDTA-blood samples were obtained by venipuncture and plasma was separated by one centrifugation step at 2000 × g for 10 minutes at room temperature (RT). All plasma samples were RNA spiked in with 3.5 μL of synthetic *Caenorhabditis elegans* miR-39 (cel-miR-39, 10 nM) to validate the efficiency of RNA extraction. Then, RNA was extracted using QIAzol Lysis Reagent (Qiagen) in combination with miRNeasy mini kit (Qiagen). One mL QIAZOL was added to 200 μL of plasma and homogenized by vigorous mixing on a vortex (15 seconds) and then incubated at RT for 5 minutes. Next, 200 μL Chloroform were added and mixed on a vortex for 10 seconds followed by incubation at RT for 2 minutes. Samples were then centrifuged at 12000 × g and 4°C for 15 minutes. The upper (aqueous) phase was transferred to a new RNase-free tube and 20 μL Glycogen (Ambion) plus 1.5 vol. ethanol were added. After that, the protocol was performed using the miRNeasy mini kit (Qiagen) and according to the manufacturer's instructions. Briefly, 750 μL of the samples were transfered to a RNeasy Mini Spin Column, centrifuged at 13000 × g for 30 seconds at RT and the flow through was discarded. This step was repeated with the remaining sample and then the column was washed once with 700 μl RWT buffer and next 3 times with RPE buffer.

Finally, RNA was eluted by adding 30 μl of DNase/RNase-free water to the column and centrifuged at 13000 × g for 1 minute at RT. Eluted RNA samples were stored at −80°C until use. Quantitation of cel-miR-39 by qRT-PCR was performed in quadruplicate using human TaqMan MicroRNA Assay Kits (Applied Biosystems) on a Corbett Rotorgene rotorcycler (Qiagen, Germany) (Supplemental figure 1a).

### Isolation of RNA from cells

RNA was isolated from cells using Trizol-chloroform extraction followed by isopropanol precipitation of the aequeous phase and centrifugation at 12.000 × g for 15 minutes at 4°C. Precipitated total RNA was washed once with 70% Ethanol and dried prior to resuspension in 30 μl nuclease-free water. RNA concentration was determined using a NanoDrop 1000 spectrophotometer (Thermo Scientific) and stored at −80°C.

### Real-time qPCR analysis of circulating microRNAs

miRNA expression profiling was performed by Exiqon Inc., Denmark using an LNA qPCR Array platform and the commercially available serum/plasma miRNA focus panel in 384-well format.

In order to evaluate the reverse transcription efficiency, prior to the reverse transcription step synthetic RNA oligonucleotides (RNA spike-in, UniSp6) were added to all samples (Supplemental figure 1b). Then, 15 μl RNA were reverse transcribed in 75 μl volume reactions using the miRCURY LNA™ Universal RT miRNA PCR, Polyadenylation and cDNA synthesis kit (Exiqon). At this step, a synthetic DNA spike-in (UniSp3) was added to all the cDNA samples (Supplemental figure 1b). Subsequently, cDNA was diluted and assayed in 10 μl PCR reactions according to the protocol for miRCURY LNA™ Universal RT miRNA PCR Human serum/plasma focus panel. Negative controls excluding template from the reverse transcription reaction were performed and profiled similar to the samples. qPCR amplifications were performed in 384-well plates, in a LightCycler 480 Real-Time PCR System (Roche). The determination of Crossing point (Cp) and melting curve analysis were done using the Roche LC software and the 2^nd^ derivative method.

In order to evaluate whether the samples were affected by haemolysis^46^, the differences between the expression of hsa-miR-23a and hsa-miR-451 were calculated. A cutoff of 8 was imposed according to the literature, with ddCp values <8 and >8 indicating low or high risk of sample contamination by haemolysis, respectively^47^ (Supplemental figure 1c). In order to assess the state of liver toxicity in all patient RNA samples, hsa-miR-122 expression levels were evaluated and log2 fold change against hsa-miR-23a was calculated (Supplemental figure 1d).

#### Data analysis

All the amplified miRNAs were analyzed for distinct melting curves and the Tm was checked to be within known specifications for the assay. Furthermore, only assays detected with 5 Cp's less than the negative control, and with Cp < 37 were included in the data analysis.

Normalization was performed using the average of miRNAs detected in all samples, an approach that has previously been reported to produce reliable results^48^. Additionally, the normfinder algorithm^49^ confirmed that the stability of the average Cp-value of the 96 miRNAs is higher than the stability of any single miRNA in the data set (see Supplemental table 3 for details). Normalized Cp values (dCp) were calculating using the follow formula: 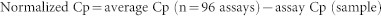


Where a higher value indicates that a determined miRNA is more abundant in the analyzed sample.

#### Power analysis

A basic power analysis calculation was performed, to assess the control of type-II errors during such a high-throughput approach to miRNA screening in plasma samples. Therefore, the distribution of standard deviations of all 175 miRNAs that were included was analyzed (Supplemental Figure 4A). The median SD amounted to 1.67. This value was chosen for calculation of sample sizes that are required to detect a certain size effect (delta Ct difference between two groups) depending on Power and significance level α (kept constant at 0.05). The results are shown as line plot and table in Supplemental Figure 4 (B,C).

### Real-time qPCR analysis of cellular microRNAs

Two μl of total RNA that had been diluted to 5 ng/μl were reverse transcribed using the Universal cDNA Synthesis Kit together with UniSp6 spike-in control to monitor the presence of enzyme inhibitors. Real-time qPCR reactions were performed in 10 μl reaction volumes in triplicates using SYBR Green Mix (Exiqon Inc.) together with commercially available primer assays for hsa-miR-146a, hsa-miR-29b-3p and hsa-miR-33a-5p and U6 and 5S rRNA as reference RNAs, respectively. PCR conditions were 95°C for 10 minutes, 45 cycles of denaturation (95°C, 10 s) and annealing/elongation (60°C, 60 s), and melting curve analysis on an LC 480 (Roche). Cp-values were calculated using the 2^nd^ derivative method and ddCt analysis was performed to calculate log_2_-fold differences between control- and mRNA-transfected samples.

### *In vitro* experiments

#### miRNA overexpression

MT-2 T-cell line was obtained from the AIDS Research and Reference Reagent program (AIDS Division, National Institute of Allergy and Infectious Disease, National Institutes of Health). For miRNA transfection, 3 × 10^4^ cells per well were seeded on a 24-well plate and transfected with 25 nM pre-miRs precursors (hsa-miR-29b-3p, hsa-miR-146a-5p, hsa-miR-33a-5p) (Ambion) or Pre-miR miRNA Negative Control (non-targeting miR control) (Ambion) using siPort NeoFx transfection reagent (Invitrogen).

#### Transfection of primary CD4-positive T cells

CD4-positive T cells were isolated from PBMCs with magnetic beads from Miltenyi (Cat.# 130-091-301) as recommended by the manufacturer. Immediately after isolation, cells (1 × 10^6^/reaction) were transfected with 150 nM pre-miRs precursors (hsa-miR-29b-3p, hsa-miR-146a-5p, hsa-miR-33a-5p) (Ambion) or hsa-miR-10a-5p (a non-functional control) (Ambion) using an Amaxa Human T cell Nucleofector Kit (Lonza VPA 1002) as recommended by the manufacturer with the transfection protocol U-014. Transfection efficiency was optimized using Cy-3 labeled non-targeting controls. Efficiencies of 62% positive cells were achieved (Supplemental Figure 4).

#### HIV in vitro infection

The HIV-NL4.3 viral strain was obtained from the NIH (AIDS-directed Program). Forty eight hours after transfection, MT-2 cells were infected with 2 ul of the viral stock (multiplicity of infection 2.8 × 10^4^) and incubated for 3 h hours at 37°C. Alternatively IL-2 and PHA- stimulated primary CD4-positive T cells were used for infection (multiplicity of infection 3 × 10^5^). Then, the cells were washed and resuspended in RPMI medium supplemented with 10% FBS. MT-2 cell culture supernatants were harvested at days 3, 6 and 9 post-infection, primary CD4 T-cells on days 4, 8, 11 and 14. The replication kinetics were examined by measuring the levels of p24 using an ELISA kit (provided by Polymun) as described recently^50^.

#### Flow cytometry

MT-2 cells were seeded in RPMI medium (supplemented with 10% FBS) in a concentration of 3 × 10^5^ cells per well on a 24-well plate and transfected with pre-miRs precursor hsa-miR-146a-5p (Ambion) or pre-miRmiRNA negative Control (non-targeting miR control) (Ambion) according to protocol described above. After 24 hour-incubation at 37°C, 5% CO_2_ the cells were transferred into FACS tubes, washed and incubated with staining antibodies against CXCR4 (BD PE mouse anti-human CD184, Lot: 54058) for 30 minutes in dark at 4°C. After another washing step the cells were resuspended in 500 μl PBS and analyzed for expression of CXCR4 using BD Biosciences FACS Canto II cytometer.

### Statistical analysis

#### Descriptive analysis

Heatmap, unsupervised hierarchical clustering and principal component analysis (PCA) were used to visualize patterns in the data set. Heatmap was produced using Euclidean distance and the hierarchical clustering by complete linkage using the heatmap.2 function in R/Bioconductor^51^. Heatmap and unsupervised hierarchical clustering were performed on the 96 miRNAs expressed in all the samples, while the PCA was performed on all the samples.

#### Inferential analysis

Chi Square test was used for evaluating differences in gender distribution between the groups. For studying the differences in CD4+ T cell levels and known time of infection between EC and CH, t-test was used. For analyzing the statistical significance of the infection assay and comparing dCp and age between groups one-way ANOVA followed by Bonferroni multiple-comparison post-hoc test were performed. Additionally, the false discovery rate (FDR) adjustment was used to control the error rate of the relative miRNAs expression. The FDR (q-values) ≤ 0.05 were considered significant^52^. Pearson correlation was used to determine the magnitude of association between the dCp and T CD4 + cell counts. The p-values ≤ 0.05 were considered significant.

## Author Contributions

R.R., L.N., S.S., H.M. and S.H. planned and performed experiments, analyzed data and wrote or corrected the manuscript. L.N. and C.P. recruited the donors and corrected the manuscript. MR. performed experiments. T.G. and C.M. collected clinical data and corrected the manuscript. Q.J. corrected the manuscript. W.R. performed experiments. G.V.R. and J.G. planned experiments, interpreted the data, supervised the project, and corrected the manuscript.

## Supplementary Material

Supplementary Information Supplemental Info

Supplementary Information Dataset 1

## Figures and Tables

**Table 1:** Donors characteristics

	Chronic HIV	ELITE Controllers	Healthy donors
**n**	9	9	6
**Gender** (male:female)	4:5	3:6	2:4
**Age** (years)	42.9 ± 7.5*	42.1 ± 7.7*	35.6 ± 13.8*
**CD4+ T cell count**	447.8 ± 227.7*	630 ± 159.5*	NA
**Viral load** (copies/ml)	<50[Fn t1-fn3]	<50[Fn t1-fn3]	X
**CD8+ T cell count**	NA	835.4 ± 549.1*	NA
**Known time of infection** (years)	8.4 ± 4.9*	7.4 ± 4.3*	X
**Coinfections** (n)^#^	3	0	X
**Opportunistic infections** (n)	3	0	X
**ART** (n)	7/8	0	X

X: not applicable, NA: not available,

*mean ± SD,

median # Hepatitis C or B virus, ART: Antiretroviral therapy.

**Table 2:** Differentially-expressed miRNAs after applying Benjamini-Hochberg correction

	Log_2_ expression*			Significance (q value)		
Name	Chronic	Elite	Normal	CH vs. EC	CH vs. HD	EC vs. HD
hsa-miR-33a-5p	−2.381	−1.073	−1.119	0.030	0.024	NS
hsa-miR-146a-5p	−0.309	0.732	0.340	0.053	NS	NS
hsa-miR-660-5p	−3.976	−4.620	−5.102	NS	0.016	NS
hsa-miR-151a-5p	1.201	1.529	1.849	NS	0.017	NS
hsa-miR-29b-3p	−2.890	−2.111	−2.204	0.030	0.029	NS
hsa-miR-28-5p	−1.008	−0.782	−0.368	NS	0.009	NS
hsa-miR-191-5p	1.341	2.012	2.171	NS	0.039	NS
hsa-miR-181a-5p	0.160	1.060	1.730	NS	0.029	NS
hsa-miR-18b-5p	−0.344	0.202	0.435	NS	0.047	NS
hsa-miR-126-3p	4.564	5.038	5.150	NS	0.017	NS
hsa-miR-423-3p	0.744	1.331	1.783	NS	0.033	NS
hsa-miR-18a-5p	−1.009	−0.380	−0.302	NS	0.049	NS
hsa-let-7i-5p	−0.152	0.158	0.569	NS	0.029	NS
hsa-miR-19b-3p	4.561	4.535	3.899	NS	0.016	NS
hsa-miR-342-3p	−0.013	−0.864	−1.557	NS	0.029	NS
hsa-miR-424-5p	−0.112	−0.633	−1.092	NS	0.029	NS
hsa-miR-16-5p	4.808	4.410	4.077	NS	0.017	NS

*dCp: normalized crossing point (log_2_ miRNA expression).

q Value: Significance calculated from p values of Post-hoc Bonferroni using Benjamini – Hochberg false discovery rate test.

NS: not significant (q > 0.05).
