# Supplementary material for: MicroRNAs differentially present in the plasma of HIV elite controllers reduce HIV infection in vitro
Source: Sci Rep. 2014 Aug 1;4:5915. doi: 10.1038/srep05915 (PMC4118195; doi:10.1038/srep05915)
Supplement: Supplementary Information — Supplemental Info [file srep05915-s1.pdf]

Supplementary Information

to the manuscript entitled:

MicroRNAs differentially present in the plasma of HIV elite controllers reduce HIV infection *in vitro*

Author List:

Reynoso Rita[1], Laufer Natalia[2,3,6,7], Hackl Matthias[1,4], Susanna Skalicky[5], Monteforte Rossella[1], Turk Gabriela[2,3], Carobene Mauricio[2,3], Quarleri Jorge[2,3], Cahn Pedro[6,7], Werner Roland[8], Stoiber Heribert[8], Grillari-Voglauer Regina[1,5], Grillari Johannes[1,5,*].

Affiliations:

[1] Department of Biotechnology, BOKU - University of Natural Resources and Life Sciences Vienna, Muthgasse 18, A-1190 Vienna, Austria.

[2] Instituto de Investigaciones Biomédicas en Retrovirus y SIDA (INBIRS).

[3] CONICET, Argentina.

[4] TAmiRNA GmbH, Muthgasse 11, A-1190 Vienna, Austria

[5] Evercyte GmbH, Muthgasse 18, A-1190 Vienna, Austria.

[6] J.A. Fernández Hospital, Infectious Diseases Unit, Buenos Aires, Argentina.

[7] Huesped Foundation, Buenos Aires, Argentina.

[8] Division of Virology, Innsbruck Medical University, Innsbruck, Austria.

Supplemental Figure Legends:

Supplemental Figure 1. Quality control of plasma profiling. A) All plasma samples were spiked in with Cel-miR-39 and amplified after RNA isolation. Raw Cp values show that the RNA extraction efficiency was similar in all the samples. Error bars indicate the standard deviation of the technical quadruplicates B) RNA spike-in (UniSp6) and DNA spike-in (UniSp3) controls were added to the samples in order to evaluate the RT reaction and qPCR amplification respectively. The graphic show that both reverse transcription and qPCR were successful. The samples EC 4 and EC 9 were re-run due to failed cDNA synthesis and the re-run was done using another spike-in dilution. C) miR-23a and miR-451 were amplified with the aim to evaluate plasma contamination due to haemolysis. A difference (dCp) lower than 8 is considered low risk of sample alteration by haemolysis. D) Differences between the expression of hsa-miR-23a and hsa-miR-122 were calculated in order to detect sample alteration due to liver toxicity. The miRNAs hsa-miR-122 and hsa-miR-194 have been associated to liver toxicity. The sample from donor CH4 showed extremely high levels of both miRNAs, affecting the profiling, therefore this sample was not considered for the analysis.

Supplemental Figure 2. Expression levels of hsa-miR-29b-3p, hsa-miR-33a-5p and hsa-miR-146a-5p in plasma from patients with progressive HIV disease and without ART. Twenty new samples were recruited and analyzed by qPCR (individual qPCR assays). miR-23a was analyzed as internal reference gene. A) As control, the samples the healthy donors were reanalyzed. As shown the repeated analysis correlated well with the previous analysis. B-D) Scatterplots showing normalized miRNA expression levels in healthy donors compared to untreated HIV samples. Non-paramteric t-tests (Mann Whitney U) were used for differential expression analysis.

Supplemental Figure 3. Transfection efficiency of CD4 T-cells with microRNA mimics. T-cells were either mock transfected using nucleofection or transfected with 150 nM Cy-3 labelled miRNA mimic to asses transfection efficiency.

Supplemental Figure 4. Power Calculations for multiplex screening of circulating microRNAs. A) The distribution of standard deviations obtained from 175 miRNAs across 24 samples was analyzed. The median standard deviation was 1.67. B) Using Statmate 2.0, size effects (Ct-Values differences between group means) for a given significance level of α=0.05 were calculated depending on the power (control of type-II error) and sample size. C) Tabular information underlying the plot in B)

Supplemental Figure 1

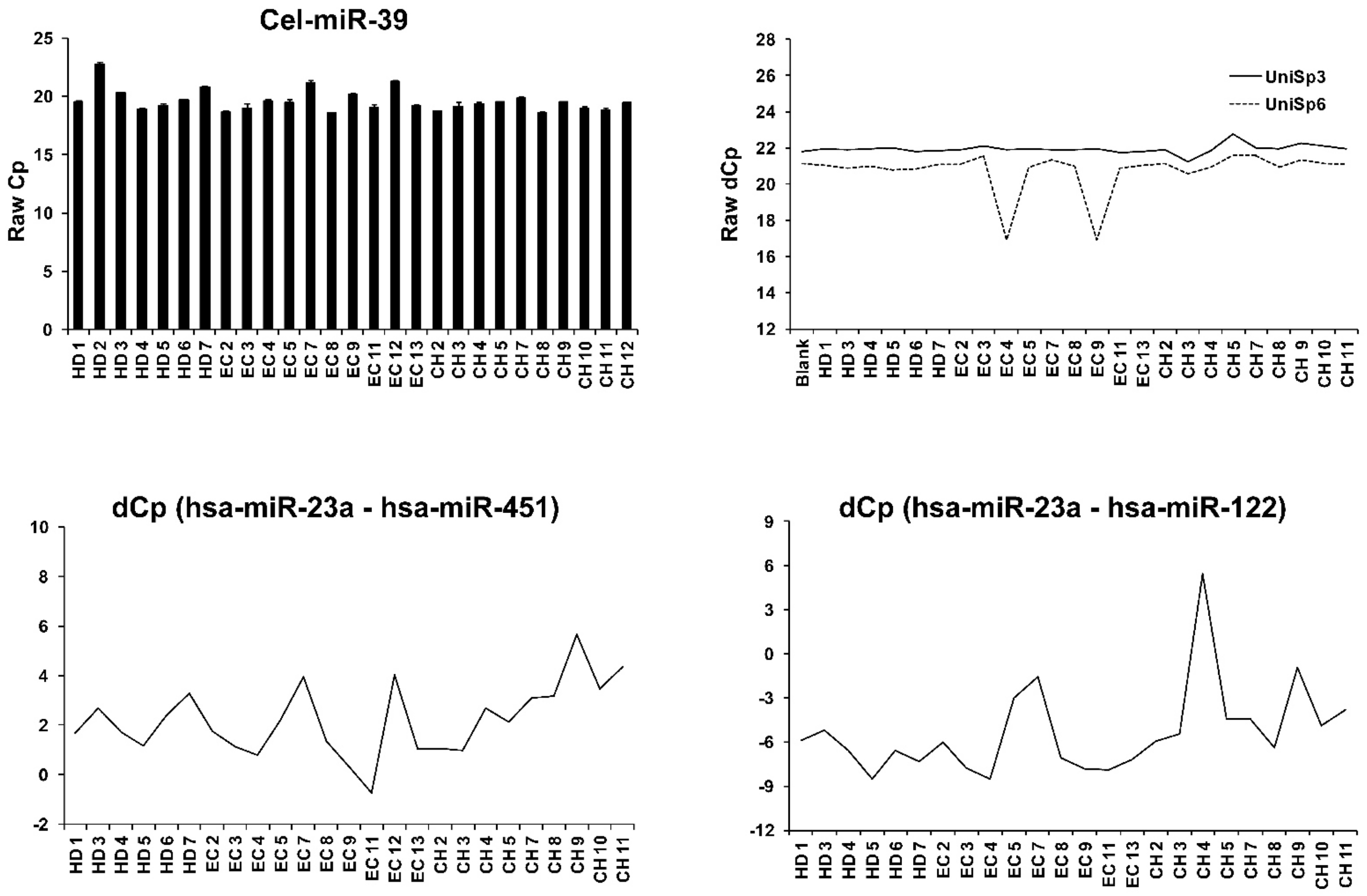

Supplemental Figure 2

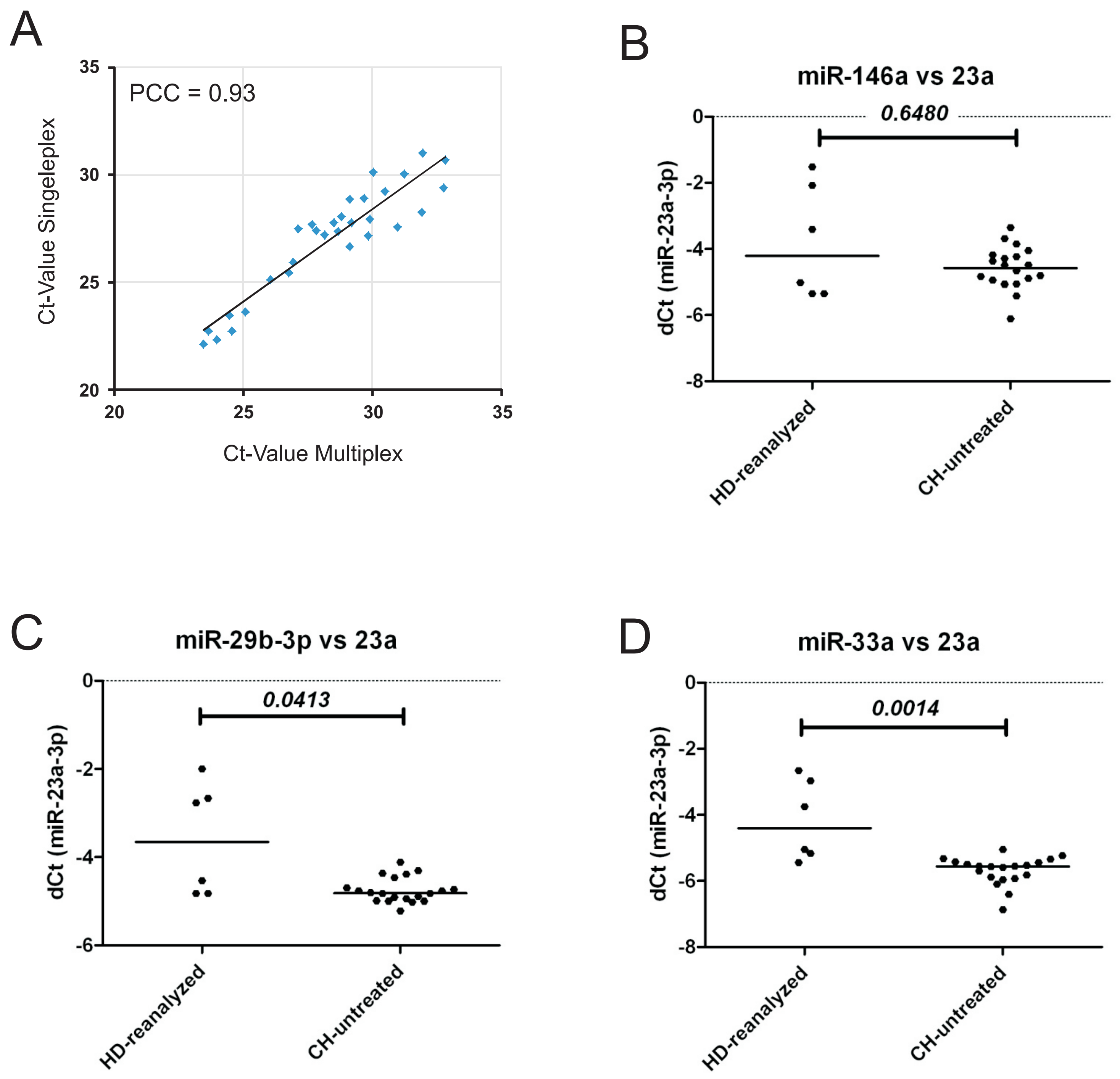

Supplemental Figure 3

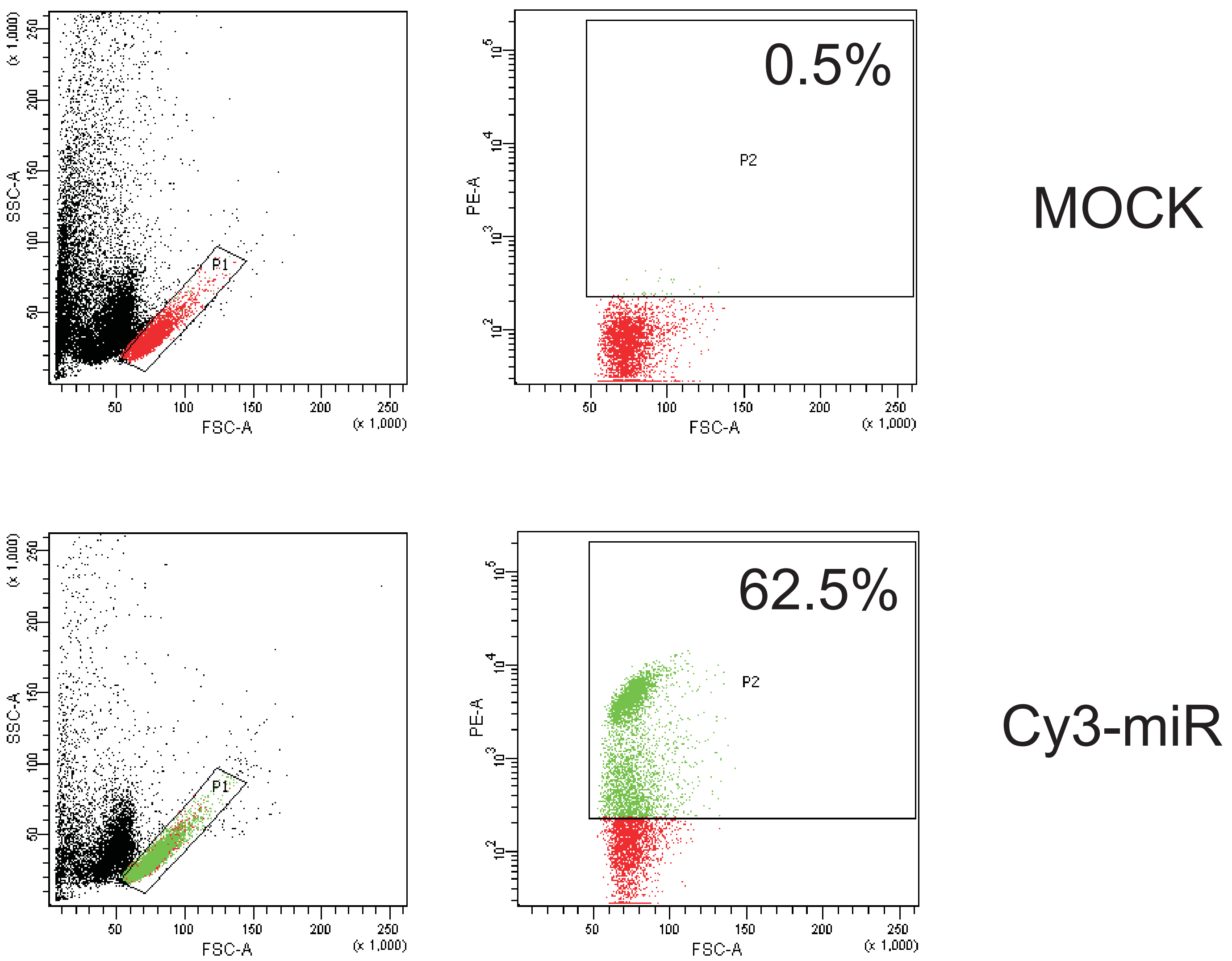

Supplemental Figure 4

A

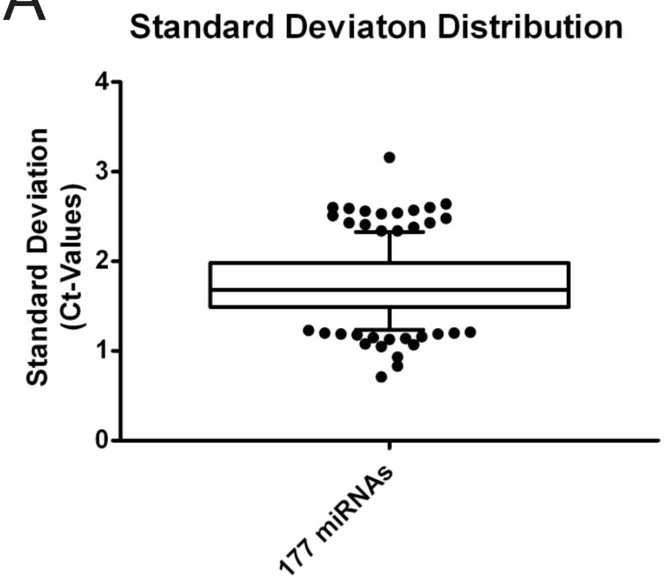

B

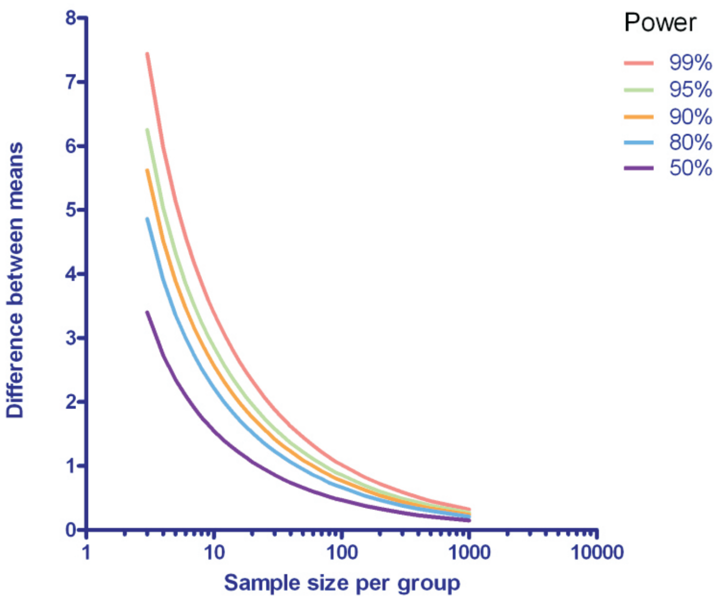

C

	Power				
**N per group**	**99%**	**95%**	**90%**	**80%**	**50%**
3	7.44	6.25	5.62	4.86	3.4
4	5.98	5.03	4.52	3.91	2.73
5	5.14	4.33	3.89	3.36	2.35
6	4.58	3.85	3.47	3	2.1
7	4.17	3.51	3.16	2.73	1.91
8	3.86	3.24	2.92	2.52	1.76
9	3.6	3.03	2.73	2.36	1.65
10	3.39	2.86	2.57	2.22	1.55
12	3.07	2.58	2.32	2	1.4
14	2.82	2.37	2.13	1.84	1.29
16	2.62	2.21	1.98	1.71	1.2
18	2.46	2.07	1.86	1.61	1.13
20	2.33	1.96	1.76	1.52	1.06
25	2.07	1.74	1.57	1.35	0.95
30	1.88	1.58	1.42	1.23	0.86
35	1.74	1.46	1.31	1.14	0.79
40	1.62	1.36	1.23	1.06	0.74
50	1.45	1.22	1.09	0.95	0.66
60	1.32	1.11	1	0.86	0.6
70	1.22	1.03	0.92	0.8	0.56
80	1.14	0.96	0.86	0.74	0.52
90	1.07	0.9	0.81	0.7	0.49
100	1.02	0.86	0.77	0.67	0.47
150	0.83	0.7	0.63	0.54	0.38
200	0.72	0.6	0.54	0.47	0.33
300	0.59	0.49	0.44	0.38	0.27
400	0.51	0.43	0.38	0.33	0.23
500	0.45	0.38	0.34	0.3	0.21
1000	0.32	0.27	0.24	0.21	0.15

Supplemental Table 1: Statistically significant differentially-expressed miRNAs from contrasts by ANOVA

		CH vs. EC			CH vs. HD			EC vs. HD		
Name	ANOVA	Difference	Post-hoc Bonferroni (p value)	FDR (q value)	Difference	Post-hoc Bonferroni (p value)	FDR (q value)	Difference	Post-hoc Bonferroni (p value)	FDR (q value)
hsa-miR-33a-5p	0.000	-1.308	0.000	0.030	-1.262	0.002	0.024	0.045	1.000	NS
hsa-miR-146a-5p	0.002	-1.041	0.001	0.053	-0.649	0.145	NS	0.392	0.306	NS
hsa-miR-151a-3p	0.008	-0.612	0.009	0.081	-0.505	0.068	NS	0.106	1.000	NS
hsa-miR-32-5p	0.309	0.778	0.986	NS	1.288	0.414	NS	0.510	1.000	NS
hsa-let-7e-5p	0.001	0.744	0.006	0.068	-0.263	1.000	NS	-1.008	0.003	0.099
hsa-miR-660-5p	0.001	0.644	0.033	0.208	1.126	0.000	0.016	0.482	0.158	NS
hsa-miR-425-3p	0.017	-0.686	0.063	NS	-0.835	0.025	0.108	-0.148	1.000	NS
hsa-miR-151a-5p	0.001	-0.328	0.087	NS	-0.648	0.001	0.017	-0.320	0.100	NS
hsa-miR-29b-3p	0.000	-0.779	0.000	0.030	-0.686	0.003	0.029	0.094	1.000	NS
hsa-miR-28-5p	0.000	-0.226	0.079	NS	-0.639	0.000	0.009	-0.414	0.012	0.301
hsa-let-7b-3p	0.003	1.221	0.002	0.060	0.720	0.245	NS	-0.501	0.237	NS
hsa-miR-199a-5p	0.004	-0.792	0.004	0.067	-0.670	0.033	0.113	0.122	1.000	NS
hsa-miR-191-5p	0.002	-0.671	0.009	0.081	-0.830	0.006	0.039	-0.159	1.000	NS
hsa-miR-590-5p	0.001	-0.468	0.004	0.066	0.000	1.000	NS	0.467	0.003	0.099
hsa-miR-181a-5p	0.004	-0.900	0.094	NS	-1.570	0.004	0.029	-0.670	0.294	NS
hsa-miR-18b-5p	0.006	-0.547	0.053	0.290	-0.780	0.007	0.047	-0.233	0.761	NS
hsa-miR-338-3p	0.018	-1.217	0.111	NS	-1.766	0.020	0.092	-0.548	0.943	NS
hsa-miR-126-3p	0.000	-0.474	0.004	0.066	-0.586	0.001	0.017	-0.111	0.848	NS
hsa-let-7d-3p	0.003	-0.378	0.005	0.068	-0.102	1.000	NS	0.276	0.020	0.380
hsa-miR-423-3p	0.005	-0.586	0.119	NS	-1.039	0.004	0.033	-0.452	0.283	NS
hsa-miR-18a-5p	0.003	-0.629	0.011	0.084	-0.707	0.008	0.049	-0.078	1.000	NS
hsa-let-7i-5p	0.003	-0.310	0.806	NS	-0.720	0.003	0.029	-0.410	0.027	0.444
hsa-miR-19b-3p	0.000	0.026	1.000	NS	0.661	0.000	0.016	0.636	0.001	0.083

hsa-miR-584-5p	0.042	-0.976	0.132	NS	-1.212	0.054	0.147	-0.236	1.000	NS
hsa-miR-186-5p	0.023	-0.773	0.095	NS	-1.065	0.030	0.112	-0.291	1.000	NS
hsa-miR-221-3p	0.012	-0.668	0.026	0.174	-0.705	0.033	0.113	-0.037	1.000	NS
hsa-miR-30e-5p	0.002	0.015	1.000	NS	0.474	0.018	NS	0.459	0.002	0.099
hsa-miR-21-5p	0.031	-0.425	0.077	NS	-0.482	0.061	NS	-0.057	1.000	NS
hsa-miR-342-3p	0.003	0.851	0.072	NS	1.543	0.002	0.029	0.693	0.267	NS
hsa-miR-652-3p	0.008	-0.566	0.008	0.081	-0.468	0.083	NS	0.098	1.000	NS
hsa-miR-324-5p	0.042	-1.107	0.388	NS	-1.869	0.040	0.127	-0.762	0.397	NS
hsa-miR-92a-3p	0.038	0.504	0.039	0.234	0.365	0.278	NS	-0.139	1.000	NS
hsa-miR-122-5p	0.036	2.533	0.156	NS	3.313	0.048	0.142	0.780	1.000	NS
hsa-miR-93-5p	0.057	-0.407	0.099	NS	-0.416	0.140	NS	-0.009	1.000	NS
hsa-miR-502-3p	0.078	0.789	0.081	NS	0.526	0.521	NS	-0.263	1.000	NS
hsa-miR-451a	0.006	1.289	0.009	0.081	1.198	0.029	0.112	-0.091	1.000	NS
hsa-let-7a-5p	0.052	1.356	0.087	NS	1.378	0.133	NS	0.021	1.000	NS
hsa-miR-148b-3p	0.047	-0.349	0.044	0.254	-0.246	0.472	NS	0.103	1.000	NS
hsa-miR-22-3p	0.029	-0.937	0.071	NS	-1.108	0.056	0.147	-0.171	1.000	NS
hsa-miR-30c-5p	0.052	1.357	0.191	NS	1.821	0.068	NS	0.465	1.000	NS
hsa-miR-136-5p	0.013	-0.709	0.627	NS	-2.059	0.011	0.065	-1.350	0.128	NS
hsa-miR-150-5p	0.019	0.797	0.475	NS	2.145	0.016	0.086	1.348	0.235	NS
hsa-miR-424-5p	0.004	0.521	0.060	NS	0.979	0.004	0.029	0.459	0.415	NS
hsa-miR-144-3p	0.005	1.170	0.004	0.066	0.651	0.145	NS	-0.519	0.629	NS
hsa-miR-185-5p	0.060	-0.836	0.294	NS	-1.237	0.068	NS	-0.401	1.000	NS
hsa-miR-16-5p	0.001	0.398	0.021	0.148	0.731	0.001	0.017	0.333	0.348	NS
hsa-miR-148a-3p	0.029	0.599	1.000	NS	1.265	0.031	0.112	0.666	0.115	NS
hsa-miR-34a-5p	0.047	1.544	0.065	NS	1.485	0.174	NS	-0.060	1.000	NS
hsa-miR-376a-3p	0.021	-0.431	1.000	NS	-1.578	0.020	0.092	-1.147	0.116	NS
hsa-miR-20a-3p	0.166	0.516	0.634	NS	0.796	0.200	NS	0.280	1.000	NS
hsa-miR-145-5p	0.035	0.086	1.000	NS	-1.427	0.148	NS	-1.513	0.037	0.538

hsa-miR-130a-3p	0.159	-0.550	0.176	NS	-0.321	0.945	NS	0.229	1.000	NS
hsa-miR-326	0.214	-0.888	0.898	NS	-1.324	0.255	NS	-0.436	1.000	NS
hsa-miR-19a-3p	0.032	0.120	1.000	NS	1.010	0.040	0.127	0.890	0.086	NS
hsa-miR-423-5p	0.118	-0.759	0.460	NS	-1.167	0.141	NS	-0.408	1.000	NS
hsa-miR-425-5p	0.096	-0.337	0.603	NS	-0.554	0.101	NS	-0.217	0.830	NS
hsa-miR-200c-3p	0.085	-0.430	0.293	NS	-0.609	0.110	NS	-0.179	1.000	NS
hsa-miR-22-5p	0.010	-0.003	1.000	NS	1.035	0.031	0.112	1.038	0.014	0.301
hsa-miR-24-3p	0.083	-0.142	1.000	NS	0.363	0.269	NS	0.505	0.093	NS
hsa-miR-23a-3p	0.092	-0.351	1.000	NS	0.393	0.370	NS	0.744	0.098	NS
hsa-let-7d-5p	0.183	0.707	0.263	NS	0.633	0.477	NS	-0.074	1.000	NS
hsa-miR-486-5p	0.022	0.806	0.021	0.148	0.509	0.189	NS	-0.297	1.000	NS
hsa-miR-29b-2-5p	0.053	-0.641	0.423	NS	-1.324	0.053	0.147	-0.683	0.612	NS
hsa-miR-142-3p	0.171	-0.215	1.000	NS	-0.410	0.205	NS	-0.195	0.492	NS
hsa-miR-205-5p	0.080	1.246	0.242	NS	-0.604	1.000	NS	-1.850	0.123	NS
hsa-miR-106b-5p	0.247	-0.312	0.386	NS	-0.080	1.000	NS	0.233	0.605	NS
hsa-miR-125a-5p	0.229	-0.050	1.000	NS	-0.531	0.435	NS	-0.481	0.346	NS
hsa-miR-335-5p	0.134	0.062	1.000	NS	0.761	0.229	NS	0.699	0.221	NS
hsa-miR-23b-3p	0.161	0.351	1.000	NS	0.886	0.213	NS	0.535	0.373	NS
hsa-miR-20a-5p	0.072	0.277	1.000	NS	0.796	0.073	NS	0.519	0.344	NS
hsa-miR-127-3p	0.030	-0.047	1.000	NS	-1.371	0.050	0.144	-1.323	0.056	0.691
hsa-miR-409-3p	0.117	-0.387	1.000	NS	-1.093	0.124	NS	-0.706	0.518	NS
hsa-miR-30b-5p	0.195	0.952	0.744	NS	1.484	0.236	NS	0.532	1.000	NS
hsa-miR-195-5p	0.242	0.741	0.649	NS	0.939	0.358	NS	0.198	1.000	NS
hsa-miR-15b-5p	0.050	0.392	0.573	NS	1.004	0.047	0.142	0.612	0.482	NS
hsa-miR-29a-3p	0.154	0.139	1.000	NS	0.719	0.302	NS	0.580	0.225	NS
hsa-miR-154-5p	0.170	-0.705	0.671	NS	-1.197	0.203	NS	-0.492	1.000	NS
hsa-miR-30d-5p	0.243	0.095	1.000	NS	0.423	0.392	NS	0.328	0.427	NS
hsa-miR-101-3p	0.057	0.022	1.000	NS	0.534	0.270	NS	0.511	0.058	0.691

hsa-miR-30a-5p	0.403	0.869	0.581	NS	0.739	1.000	NS	-0.130	1.000	NS
hsa-miR-99b-5p	0.176	-0.009	1.000	NS	-0.657	0.398	NS	-0.648	0.234	NS
hsa-miR-143-3p	0.406	-0.316	1.000	NS	-1.039	0.681	NS	-0.723	0.767	NS
hsa-miR-17-5p	0.075	0.218	1.000	NS	0.828	0.080	NS	0.610	0.282	NS
hsa-miR-192-5p	0.459	1.076	1.000	NS	1.629	0.660	NS	0.553	1.000	NS
hsa-miR-133b	0.230	0.268	0.975	NS	-0.694	1.000	NS	-0.961	0.282	NS
hsa-miR-27b-3p	0.262	0.152	1.000	NS	0.883	0.475	NS	0.732	0.414	NS
hsa-miR-421	0.099	-0.037	1.000	NS	0.441	0.122	NS	0.477	0.273	NS
hsa-let-7i-3p	0.431	-0.418	0.911	NS	-0.529	0.722	NS	-0.112	1.000	NS
hsa-miR-301a-3p	0.285	0.033	1.000	NS	-0.527	1.000	NS	-0.560	0.352	NS
hsa-miR-629-5p	0.405	-0.491	1.000	NS	-0.992	0.566	NS	-0.502	1.000	NS
hsa-miR-128	0.516	-0.289	0.872	NS	-0.086	1.000	NS	0.203	1.000	NS
hsa-miR-223-3p	0.368	-0.374	1.000	NS	0.332	1.000	NS	0.706	0.489	NS
hsa-miR-29a-5p	0.150	0.056	1.000	NS	-0.820	0.232	NS	-0.876	0.285	NS
hsa-miR-15a-5p	0.267	0.079	0.452	NS	-0.111	1.000	NS	-0.189	0.585	NS
hsa-miR-27a-3p	0.142	0.023	1.000	NS	1.166	0.215	NS	1.143	0.265	NS
hsa-miR-210	0.244	-0.639	0.877	NS	-1.140	0.305	NS	-0.500	1.000	NS
hsa-miR-132-3p	0.452	-0.416	0.677	NS	-0.322	1.000	NS	0.095	1.000	NS
hsa-miR-194-5p	0.404	1.388	0.764	NS	1.451	0.741	NS	0.063	1.000	NS
hsa-miR-551b-3p	0.510	-0.424	0.898	NS	-0.024	1.000	NS	0.400	1.000	NS
hsa-miR-497-5p	0.280	-0.334	0.978	NS	-0.688	0.358	NS	-0.354	1.000	NS
hsa-miR-320b	0.325	-0.224	1.000	NS	-0.728	0.438	NS	-0.504	0.815	NS
hsa-miR-320a	0.347	-0.300	1.000	NS	-0.708	0.454	NS	-0.407	1.000	NS
hsa-miR-28-3p	0.016	-0.236	0.077	NS	-0.402	0.021	0.092	-0.165	1.000	NS

dCp: normalized crossing point (log_2 miRNA expression)

q Value: Significance calculated from p values of Post-hoc Bonferroni, using Hochberg test to calculate the False Discovery Rate.

NS: not significant ($q > 0.05$)

Supplemental Table 2: Flow cytometry analysis of CXCR4 in MT2 cells after transfection with hsa-miR-146a-5p.

	Mean fluorescence intensity
**Untreated cells**	179
**Isotype control**	52
**Non targeting control**	178
**hsa-miR-146a-5p**	176
